# Effect of ethanol fumigation on pericarp browning associated with phenol metabolism, storage quality, and antioxidant systems of wampee fruit during cold storage

**DOI:** 10.1002/fsn3.1617

**Published:** 2020-05-16

**Authors:** Yuanzhi Shao, Zitao Jiang, Jiaoke Zeng, Wen Li, Yu Dong

**Affiliations:** ^1^ College of Life Science and Medicine Hainan University Haikou China; ^2^ College of Horticulture Hainan University Haikou China; ^3^ Department of Horticulture Mid‐Columbia Agricultural Research and Extension Center Oregon State University Hood River OR USA

**Keywords:** antioxidant systems, ethanol, pericarp browning, storage quality, wampee fruit

## Abstract

Wampee fruit is a popular fruit cultivar in South‐East Asia due to its high levels of nutrients and antioxidants; however, pericarp browning leads to a short storage life with great economic loss during years. The purpose of this work was to determine whether postharvest ethanol fumigation affected pericarp browning development of wampee fruit during 12 days of storage at 8 ± 0.5°C, and if so, how it is related to phenol metabolism and how it affects quality attributes and antioxidant systems during storage. After fruits were fumigated with 100, 300, 500, and 800 μl/L for 5 hr at 22 ± 0.5°C, ethanol significantly reduced the development of pericarp browning by increasing total phenolics (TP) content and decreasing the activity of polyphenol oxidase (PPO), especially in 500 μl/L ethanol treatment. Additionally, ethanol delayed the losses in fruit firmness (FF), soluble solid content (SSC), and titratable acidity (TA), retarded weight loss and accumulation of malondialdehyde (MDA) content and maintained relatively high contents of ascorbic acid (AsA), total flavonoids (TF), and total antioxidant capacity (TAC) and activities of superoxide dismutase (SOD), catalase (CAT), and peroxidase (POD). In conclusion, results demonstrated that postharvest ethanol fumigation in wampee fruit has ability to reduce pericarp browning development by regulating phenol metabolism and enhancing antioxidant systems.

## Graphical abstract

Ethanol fumigation reduced the development of pericarp browning; ethanol fumigation delayed the losses in firmness, the degradation of TSS, TA, and VC of wampee fruit. Ethanol fumigation maintained high contents of AsA, TF, and TAC and activities of SOD, CAT, POD in wampee fruit.

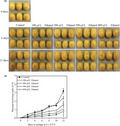

## 1 INTRODUCTION

Wampee (*Clausena lansium* (Lour.) Skeels) is a species of the Rutaceae family, widely cultivated in the south of China, such as Guangdong, Guangxi, Hainan, and Yunnan provinces (Chen, Zhang, Chen, Han, & Gao, 2017). It is famous for its nutritional value, high levels of sugar, acid, and mineral elements, and its leaves and seeds have been used in Chinese medicine for years (Li & Xing, 2016). Prasad et al. (2010) found that the peel tissue of wampee fruit was rich in amounts of coumarins, amides, and carbazole alkaloids, which were identified as antioxidant and anticancer function. However, wampee is a perishable fruit with a short storage life. Additionally, postharvest issues including softening, pericarp browning, flavor loss, and disease infection during storage or shipping negatively impact the growers' or industries' return.

Fruit appearance has been identified as an important quality trait in fruits. Consumers purchase decisions are influenced by the browning of pericarp tissue. The cause of browning developing is attributed to the formation of the brown‐pigment *o*‐quinones by catalyzing the oxidation of phenolic compounds with polyphenol oxidase (PPO) (Ioannou, 2013; Queiroz, Mendes‐Lopes, Fialho, & Valente‐Mesquita, 2008; Singh et al., 2018). Additionally, decreases of antioxidants (i.e. ascorbic acid and flavonoids) and antioxidant enzymes (i.e. superoxide dismutase, catalase, and peroxidase) may be responsible for the development of browning (Hodges, Lester, Munro, & Toivonen, 2004; Manzocco, Calligaris, Mastrocola, Nicoli, & Lerici, 2000; Zhang et al., 2015). Therefore, developing strategies to reduce pericarp browning will improve the fruit quality of wampee fruit.

Ethanol is authorized as a Generally Recognized as Safe (GRAS) substance used in food industries (Yan, Yang, & Luo, 2015). Postharvest ethanol fumigation or dipping has been demonstrated in sanitizing the surface of fresh fruits and vegetables and preventing fruit against the growth of microbial populations throughout the entire storage periods (Opio et al., 2017; Wang, Cao, Di, Liao, & Zheng, 2015). Furthermore, ethanol effectively suppressed ethylene biosynthesis, maintained high‐quality attributes, reduced the incidence of postharvest‐related disorders such as chilling injury, and eventually improved storage quality of fruits (Jin, Lv, Liu, Qi, & Bai, 2013; Yan, Luo, Zhou, & Ingram, 2017). However, little information is available on the effect of ethanol fumigation on wampee fruit and its relation to pericarp browning.

The objective of this work was firstly to investigate the effect of ethanol on pericarp browning development, phenol metabolism, and storage quality during storage and then secondly to evaluate the role of ethanol in antioxidant systems of wampee fruit.

## 2 MATERIALS AND METHODS

### 2.1 Fruit material

Wampee fruits (*Clausena lansium* (Lour.) Skeels, cv. ‘Dajixin’) were hand‐harvested from an orchard in Haikou, Hainan, China (20.02°N, 111.11°W, elevation 15 m). Fruits were selected for uniform size and color and free of any visible signs of damage or diseases. The fruits were placed into plastic containers with 1‐kg polyethylene (PE) bags (0.01 mm thickness, XingFeng Co.) and then transported to the Horticulture Postharvest Lab of Hainan University within 2 hr.

### 2.2 Treatments

A total of 4,050 wampee fruit were randomly selected and divided into five treatments, then sealed in plastic containers, and exposed to 100, 300, 500, and 800 μl/L ethanol for 5 hr at 22 ± 0.5°C. Control fruit was exposed to air under the same condition. After treatment, fruits were packed in unsealed PE bags and then stored at 8 ± 0.5°C and 90% relative humidity in refrigerators (PRX‐350D‐DN, Beijing Puxi Electric Appliance Co. Ltd) for up to 12 days (because 80% of the fruits have lost their commercial properties when stored for 12 days, we took samples until the 12th day). After 2, 4, 6, 8, 10, and 12 days, a sample of 135 fruit per treatment at each time was transferred to 22 ± 0.5°C. Forty‐five fruits were evaluated for quality attributes and antioxidant analysis and 90 fruit were used for pericarp browning index and water loss evaluation. Pericarp tissue from each treatment was quick‐frozen and grounded in liquid nitrogen and then stored at −80°C.

### 2.3 Evaluation of pericarp browning index

Pericarp browning index was evaluated from each treatment of 30 fruit per replicate and assessed by measuring the extent of total browned area on each fruit pericarp. Grading was standardized using 5‐point scale (Shao, Xie, Chen, & Li, 2013): 1, no pericarp browning; 2, 0%–25% pericarp browning area; 3, 25%–50% pericarp browning area; 4, 50%–75% pericarp browning area; 5, 75%–100% pericarp browning area. Pericarp browning index was calculated as the sum of the number of fruit in each of the five categories by the five factors 1, 2, 3, 4, and 5, and the whole divided by 30 fruit.

### 2.4 Measurement of polyphenol oxidase (PPO) activity and total phenolic (TP) content

Polyphenol oxidase activity was measured as described by Luo, Wu, Xie, and Chen (2012) with a slight modification. Two grams of pericarp tissue from 15 fruit prereplicate was extracted and homogenized in 5 ml 50 mmoL/L sodium phosphate buffer (pH 7.8) containing 0.8 g/L polyvinylpolypyrrolidone (PVPP) and 1 mmoL/L EDTA‐Na_2_. After centrifuging at 12,000 *g* for 30 min at 4°C, a sample of the extract (0.1 ml) was added to 3.0 ml 100 mmoL/L sodium phosphate buffer (pH 6.4) containing 50 mmoL/L catechol. One unit of PPO activity was defined as an increase of 0.01 in absorbance at 420 nm/min.

Total phenolics was measured as described by Habibi and Ramezanian (2017), with a slight modification. One gram of pericarp tissue from 15 fruit prereplicate was extracted and homogenized in 5 ml methanol containing 10 ml/L HCl for 20 min at 4°C in darkness, then centrifuged at 12,000 *g* at 4°C for 30 min. A sample of the extract (5 ml) was diluted to a final volume of 50 ml with methanol containing 10 ml/L HCl. The supernatant at 280 nm was recorded using a spectrophotometer (T6, New Century Inc). A standard calibration curve was constructed using gallic acid and the data were expressed on a fresh weight basis as mg gallic acid equivalent (GAE) kg^−1^.

### 2.5 Evaluations of fruit firmness (FF), soluble solids content (SSC), and titratable acidity (TA)

Fruit firmness of 15 fruit per replicate was measured at two opposite sides of the equator of each fruit after removing 2‐mm‐thick peel disks using a hand‐held hardness meter (FHM‐1, Chuk Eatate Co.) with a 12‐mm conical probe. The maximum force was recorded and expressed in newton (N). After FF determination, twenty grams of wampee fruit was juiced. SSC was determined using a refractometer (N‐1α, Atago Ltd), and data were expressed as percentage (%). TA was determined by titrating 5 ml juice and 50 ml distilled water plus three drops of phenolphthalein to pH 8.3 with 0.1 mol/L NaOH using pH meter (ST20, OHOUS Company) and expressed as the equivalent percentage of citric acid.

### 2.6 Determinations of weight loss and malondialdehyde (MDA) content

After evaluation of pericarp browning index, fruits from each treatment of 30 fruit per replicate were weighted at each time. Weight loss was expressed as percentage loss of original weight.

Malondialdehyde content was measured as described by Ding et al. (2015) with a slight modification. Two grams of pericarp tissue from 15 fruit prereplicate was homogenized in 5.0 ml 0.05 mol/L sodium phosphate buffer (pH 7.8) and centrifuged at 12,000 *g* for 15 min at 4°C. A sample of the supernatant (3.0 ml) was added to 3.0 ml 5 g/L thiobarbituric acid. After boiling for 15 min and centrifuging at 12,000 *g* for 15 min at 4°C, the absorbance was measured at 450, 532, and 600 nm. MDA content was calculated according to the formula 6.45 × (*A*_532_ − *A*_600_) − 0.56 × *A*_450_ and data were expressed on a fresh weight basis as μmol/kg.

### 2.7 Determinations of contents of ascorbic acid (AsA), total flavonoids (TF), and total antioxidant capacity (TAC)

Ascorbic acid content was measured as described by Wang et al. (2016). Two grams of pericarp tissue from 15 fruit prereplicate was homogenized in 5 ml of 20 g/L oxalic acid and centrifuged at 12,000 *g* for 15 min at 4°C. The supernatant was diluted to a final volume of 50 ml with 20 g/L oxalic acid solution. AsA content was determined by titrating 10 ml the diluted solution with 2,6‐dichlorophenolindophenol (DCPIP) solution. The volume of DCPIP solution used to reach a permanent red color was recorded. A standard calibration curve was constructed using ascorbic acid, and the data were expressed on a fresh weight basis as mg ascorbic acid equivalent (AAE) kg^−1^.

Total flavonoids content was measured as described by Habibi and Ramezanian (2017) with a slight modification. One gram of pericarp tissue from 15 fruit prereplicate was extracted and homogenized in 25 ml methanol containing 10 ml/L HCl for 20 min at 4°C in darkness and then centrifuged at 12,000 *g* at 4°C for 30 min. The supernatant at 325 nm was recorded. A standard calibration curve was constructed using catechin and the data were expressed on a fresh weight basis as mg catechin equivalent (CE) kg^−1^.

Total antioxidant capacity content was determined by 1,1‐diphenyl‐2‐picrylhydrazyl (DPPH) assay. The ability to scavenge DPPH free radicals was measured as described by Alothman, Kaur, Fazilah, Bhat, and Karim (2010). Briefly, a sample of the TF extract (100 μl) was added to 2.9 ml 0.1 mol/L DPPH. A control sample consisting of the same volume of solvent was used to measure the maximum DPPH absorbance. The absorbance at 517 nm was recorded to determine the concentration of remaining DPPH. TAC content was calculated according to the formula (*A*_517 control_ − *A*_517 sample_)/*A*_517 control_ × 100, and data were expressed on the percentage of the inhibition of DPPH radical.

### 2.8 Determinations of activities of superoxide dismutase (SOD), catalase (CAT), and peroxidase (POD)

Superoxide dismutase activity was measured as described by Shadmani, Ahmad, Saari, Ding, and Tajidin (2015) with a slight modification. Two grams of pericarp tissue from 15 fruit prereplicate was homogenized in 5 ml 50 mmol/L sodium phosphate buffer (pH 7.8) containing 0.5 g/L PVPP and 0.5 mmoL/L EDTA‐Na_2_. After centrifuging at 12,000 *g* for 20 min at 4°C, a sample of the extract (0.1 ml) was added to 2.9 ml 50 mmol/L sodium phosphate buffer (pH 7.8) containing 130 mmol/L methionine, 750 μmol/L nitro blue tetrazolium (NBT), and 20 μmol/L riboflavin. One unit of SOD activity was defined that caused 50% inhibition of the reduction of NBT as monitored per min at 560 nm.

Catalase activity was measured as described by Beers and Sizer (1952). One grams of pericarp tissue from 15 fruit prereplicate was homogenized in 5 ml 50 mmol/L sodium phosphate buffer (pH 7.8). After centrifuging at 12,000 *g* for 20 min at 4°C, a sample of the extract (0.3 ml) was added to 2.0 ml 50 mmol/L sodium phosphate buffer (pH 7.8) and 20 mmol/L H_2_O_2_. One unit of CAT activity was defined the amount of enzyme capable of causing a 0.01 change per min at 240 nm.

Peroxidase activity was measured as described by Zhang et al. (2019) with a slight modification. Two grams of pericarp tissue from 15 fruit prereplicate was homogenized in 5 ml 100 mmol/L acetate buffer (pH 5.5) containing 1 mmol/L polyethylene glycol, 40 g/L PVPP, and 10 g/L Triton X‐100. After centrifuging at 12,000 *g* for 20 min at 4°C, a sample of the extract (0.1 ml) was added to 3.0 ml of 25 mmol/L guaiacol and 0.2 ml of 0.5 mol/L H_2_O_2_. One unit of POD activity was defined the amount of enzyme capable of causing a 0.01 change per min at 470 nm.

### 2.9 Statistical analysis

Experiments were performed using a completely randomized design. One‐way analysis of variance (ANOVA) was carried out to determine the significant differences between means using Duncan's multiple range test (*p* < .05). The data were subjected to analyze using SigmaPlot software (version 10.0, Jandel Scientific).

## 3 RESULTS AND DISCUSSION

### 3.1 Effects of ethanol fumigation on pericarp browning development

**FIGURE 1:**
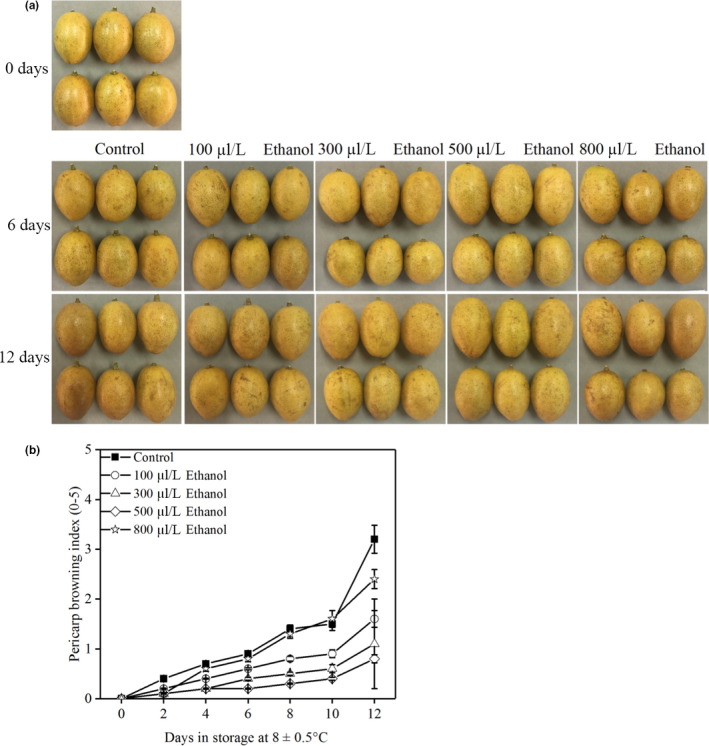
Wampee (*Clausena lansium* (Lour.) Skeels, cv. ‘Dajixin’) fruit displayed pericarp browning (a) after 0, 6, and 12 days of storage at 8 ± 0.5°C. Pericarp browning index (b) of wampee fruit treated with 100, 300, 500, and 800 μl/L of ethanol stored at 8 ± 0.5°C. Values are the mean ± standard deviation (*SD*). Different letters indicate significant differences among treatments according to Duncan's multiple range test (*p* < .05)

Pericarp browning index dramatically increased in all treatments throughout the entire 12‐d storage period (Figure 1a and b). Ethanol effectively inhibited the development of pericarp browning compared the control fruit. After 12 days of storage at 8 ± 0.5°C, the pericarp browning index of 100, 300, 500, and 800 μl/L ethanol treatments developed 1.6, 1.1, 0.8, and 2.4, respectively, indicating that the response of wampee fruit to pericarp browning was dependent on the application concentration of ethanol, as previously shown in Chinese bayberry (Zhang et al., 2007), strawberry (Li et al., 2018), and mulberry (Choosung, Utto, Boonyaritthongchai, Wasusri, & Wongs‐Aree, 2019) fruit.

### 3.2 PPO activity and TP content

**FIGURE 2:**
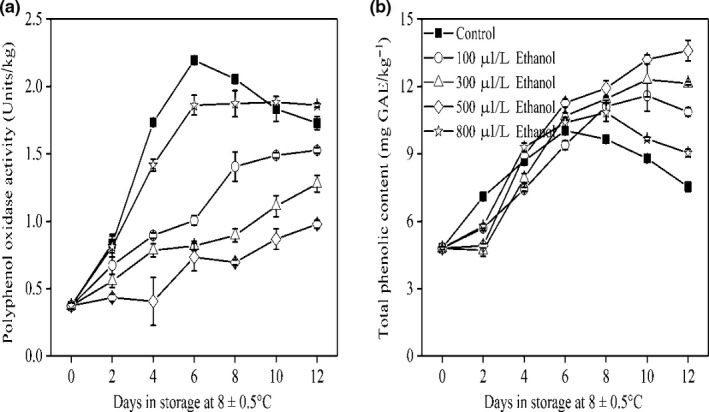
PPO activity (a) and TP content (b) of wampee fruit treated with 100, 300, 500, and 800 μl/L of ethanol stored at 8 ± 0.5°C. Values are the mean ± *SD*. Different letters indicate significant differences among treatments according to Duncan's multiple range test (*p* < .05)

It has been well documented that the changes in phenolic concentration and PPO activity result in the development of browning in fruits (Joslyn & Ponting, 1951; Sapers & Hicks, 1989). Developing an effective way to reduce the activity of PPO may directly control the browning (Laurila, Kervinen, & Ahvenainen, 1998; Moon, Young Kim, Yeul Ma, & Lee, 2019). In this study, ethanol applied at 100, 300, and 500 μl/L significantly suppressed the increase of the PPO activity during the whole storage period (Figure 2a), indicating that ethanol might change the active site or structure of PPO, resulting in a reduction of browning (Valero, Varon, & Garcia‐Carmona, 1990). The elevated TP contents were observed in all treatments during 6 days of storage (Figure 2b). Beyond 6 days, the control and 500 μl/L ethanol‐treated fruit exhibited declines of TP content, while 100, 300, or 500 μl/L ethanol‐treated fruit maintained a relatively stable or increase of TP content until 12 days, suggesting that the loss of TP in the control and 500 μl/L ethanol‐treated fruit might be due to the production of *o*‐quinones by oxidizing the phenolic compounds. Taken together, one possible explanation for controlling pericarp browning by ethanol may be attributed to reduced PPO activity and maintain TP level (Cheng, Liu, Feng, Dong, & Guan, 2019).

### 3.3 Storage quality

**FIGURE 3:**
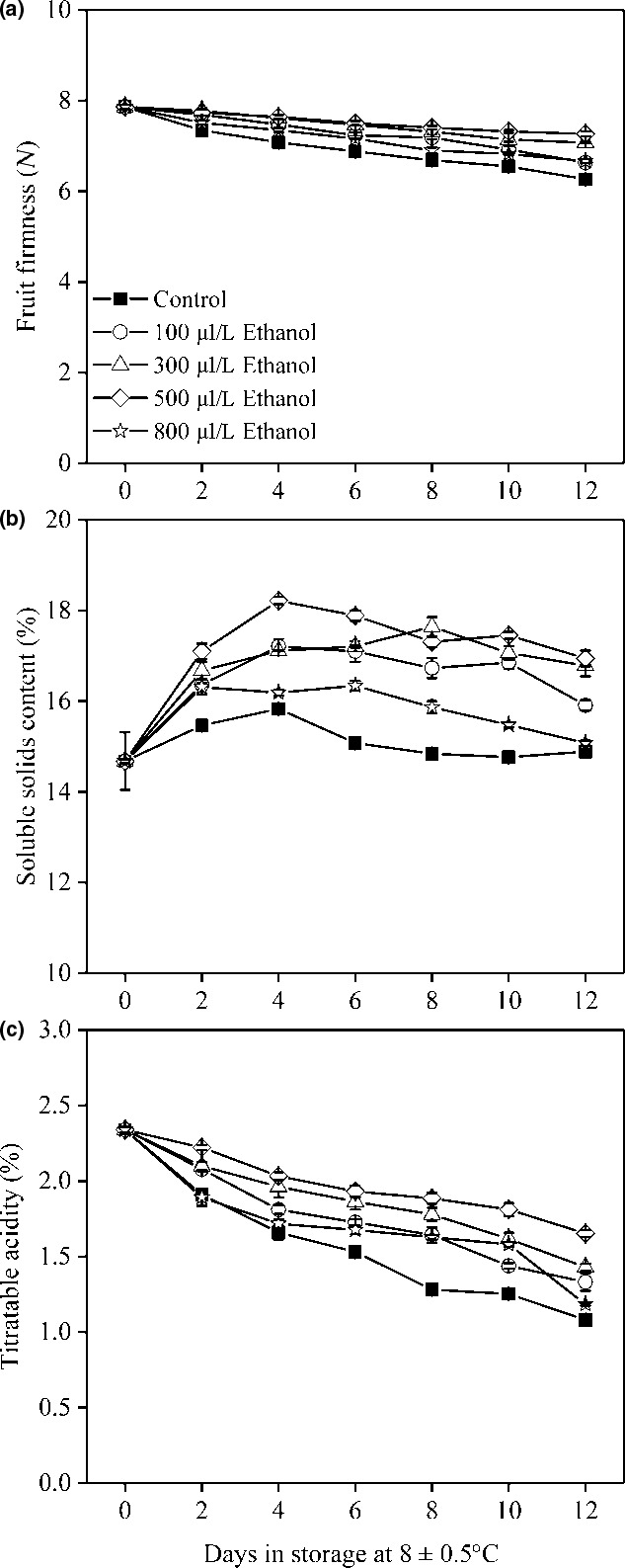
Fruit FF (a), soluble solids content (b), and titratable acidity (c) of wampee fruit treated with 100, 300, 500, and 800 μl/L of ethanol stored at 8 ± 0.5°C. Values are the mean ± *SD*. Different letters indicate significant differences among treatments according to Duncan's multiple range test (*p* < .05)

In this study, compared to the initial, the control fruit dramatically declined in FF and TA after 12 days of storage (Figure 3a and c). The increase of SSC was significantly reduced in the control treatment (Figure 3b). Ethanol slowed down the decline of FF and TA, while increased the level of SSC during storage. The effect of the 500 μl/L ethanol treatment showed the greatest positive effects on inhibiting the losses in FF and TA and stimulating the accumulation of SSC, indicating that application of ethanol at 500 μl/L was effective at significantly reducing pericarp browning and maintaining storage quality compared to the nontreated fruit. However, raising application concentration of ethanol from 500 to 800 μl/L caused increases of pericarp browning and quality deterioration, indicating that a high application concentration of ethanol might result in damage to fruit cells.

### 3.4 Weight loss and MDA content

**FIGURE 4:**
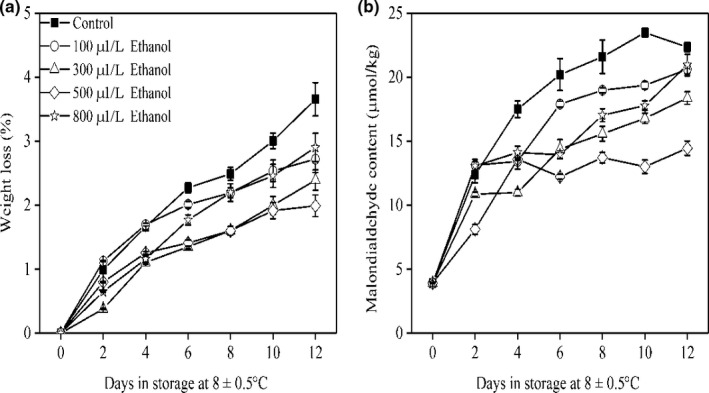
Weight loss (a) and MDA content (b) of wampee fruit treated with 100, 300, 500, and 800 μl/L of ethanol stored at 8 ± 0.5°C. Values are the mean ± *SD*. Different letters indicate significant differences among treatments according to Duncan's multiple range test (*p* < .05)

It is clear that the PPO and phenol compounds are located at the different subcellular compartments (Veltman et al., 1999); for example, PPO was found in chloroplasts and mitochondria, and phenol compounds were located at the vacuole (Yamaki, 1984). The breakdown of the cell membrane resulted more PPO met phenol compounds triggering the reaction of enzymatic browning (Dong, Liu, Zhao, Zhi, & Guan, 2015). In this study, weight loss and MDA content increased in all treatments throughout the entire storage period (Figure 4). Ethanol significantly reduced the increase of weight loss and accumulation of MDA, indicating that the ethanol had a positive effect on membrane integrity (Dong, Feng, et al., 2015). As a result, a slow developing of pericarp browning was observed in ethanol‐treated fruit. In addition, the production of reactive oxygen species (ROS) was detrimental to the cell membrane systems of wampee fruit because they accelerated the membrane lipid peroxidation and degradation of membrane structure (Fu et al., 2011; Ye, Chang, Brennan, Brennan, & Guo, 2019).

### 3.5 Antioxidants and antioxidant enzymes

**FIGURE 5:**
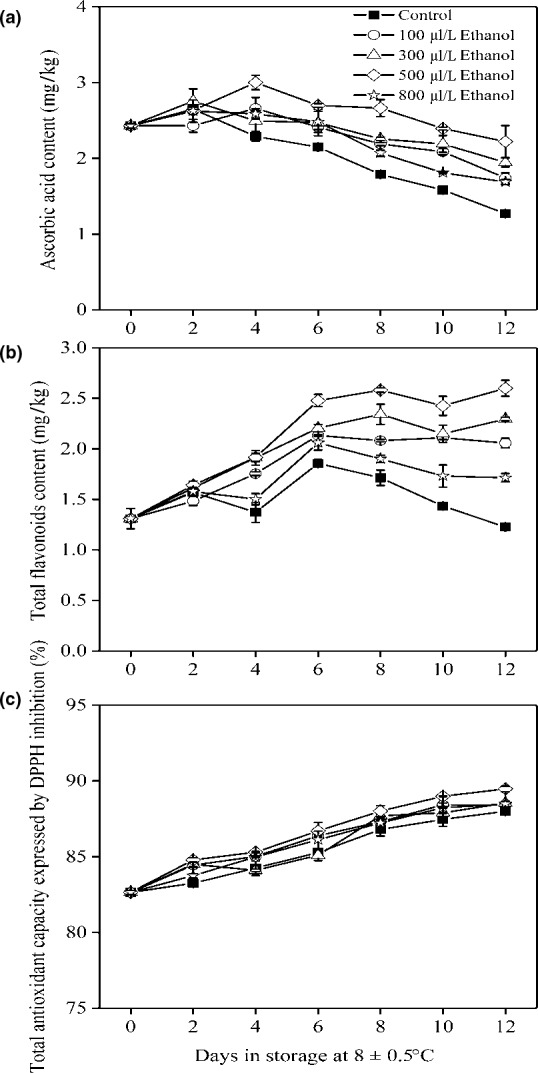
AsA content (a), TF content (b), and TAC content expressed by DPPH scavenging (c) of wampee fruit treated with 100, 300, 500, and 800 μl/L of ethanol stored at 8 ± 0.5°C. Values are the mean ± *SD*. Different letters indicate significant differences among treatments according to Duncan's multiple range test (*p* < .05)

Fortunately, fruit developed efficient ROS‐scavenging systems by nonenzymatic antioxidants (i.e. AsA and flavonoids) and antioxidant enzymes (i.e. SOD, CAT, and POD) to reduce the accumulation of ROS stabilizing cell membrane structure (Hodges et al., 2004; Shewfelt & Del Rosario, 2000). In this study, the contents of AsA and TF in the control fruit increased after 2 and 6 days of storage, respectively, and thereafter decreased (Figure 5a and b). TAC content gradually increased in all fruit during storage (Figure 5c). Ethanol delayed the loss of AsA and maintained high contents of TF and TAC than the control fruit over the entire storage period. Clearly, these greater nonenzymatic antioxidants in ethanol‐treated fruit scavenged the production of ROS and provided benefits in stabilizing membrane systems and eventually reducing the development of pericarp browning.

**FIGURE 6:**
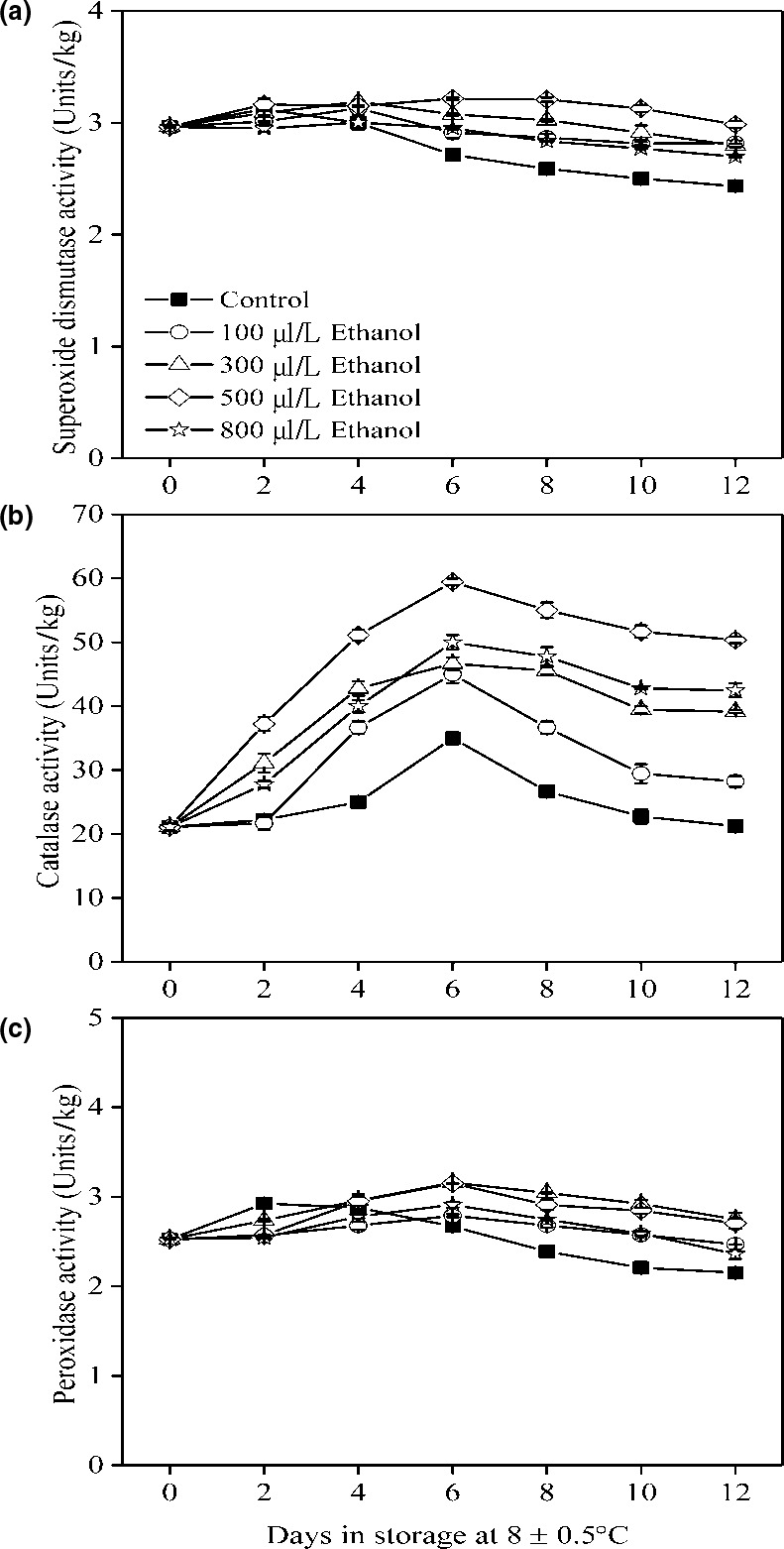
SOD activity (a), CAT activity (b), and POD activity (c) of wampee fruit treated with 100, 300, 500, and 800 μl/L of ethanol stored at 8 ± 0.5°C. Values are the mean ± *SD*. Different letters indicate significant differences among treatments according to Duncan's multiple range test (*p* < .05)

In loquat fruit, 300 μl/L ethanol fumigation inhibited anthracnose rot by maintaining high SOD activity and low activities of CAT and ascorbate peroxidase (APX) (Wang et al., 2015). In fresh‐cut strawberry, 4 ml/kg ethanol vapor improved fruit quality by enhancing the activities of SOD, CAT, and POD (Li et al., 2018). In banana, ethanol had a potential to delay decay and extend shelf life by increasing activities of SOD and CAT in pericarp tissues (De França, Braga, Laureth, Dranski, & de Andrade Moura, 2019). In this study, compared to the control fruit, ethanol‐treated fruit maintained high levels of activities of SOD, CAT, and POD during storage (Figure 6). Results indicated that the high levels of SOD, CAT, and POD in ethanol‐treated fruit could efficiently convert the ROS into water or oxygen resulting in alleviation of membrane damage (Apel & Hirt, 2004). Therefore, the control of pericarp browning was attributed to the high nonenzymatic antioxidants and antioxidant enzymes which were inactive by ethanol.

## 4 CONCLUSION

The results of the present study demonstrated that fumigation application of ethanol at 500 μl/L could be used in wampee fruit to reduce the development of pericarp browning and improve fruit quality following 12 days of storage at 8 ± 0.5°C. Ethanol suppressed the PPO activity, maintained high TP content, delayed losses in FF and TA, and inhibited the weight loss and accumulation of MDA. Additionally, high nonenzymatic antioxidants (AsA and total flavonoids) and antioxidant enzymes (SOD, CAT, and POD) in ethanol‐treated fruit had positive effects of reducing membrane lipid peroxidation and scavenging ROS damage during storage. Taken together, ethanol has the potential to control pericarp browning, enhance antioxidant systems, and extend the storability of wampee fruit.

## CONFLICT OF INTEREST

The authors declare that they have no conflicts of interest.

## ETHICAL APPROVAL

Ethics approval was not required for this research.
